# A systematic review of molecular representation learning foundation models

**DOI:** 10.1093/bib/bbaf703

**Published:** 2026-01-09

**Authors:** Bosheng Song, Jiayi Zhang, Ying Liu, Yuansheng Liu, Jing Jiang, Sisi Yuan, Xia Zhen, Yiping Liu

**Affiliations:** College of Computer Science and Electronic Engineering, Hunan University, 116 Lushan South Road, Yuelu District, 410086 Changsha, China; College of Computer Science and Electronic Engineering, Hunan University, 116 Lushan South Road, Yuelu District, 410086 Changsha, China; College of Computer Science and Electronic Engineering, Hunan University, 116 Lushan South Road, Yuelu District, 410086 Changsha, China; College of Computer Science and Electronic Engineering, Hunan University, 116 Lushan South Road, Yuelu District, 410086 Changsha, China; College of Computer Science and Electronic Engineering, Hunan University, 116 Lushan South Road, Yuelu District, 410086 Changsha, China; School of Chinese Medicine, Hong Kong Baptist University, 15 Baptist University Road, Kowloon Tong, Kowloon, Hong Kong SAR 999077, China; National Laboratory for Parallel and Distributed Processing, School of Computer, National University of Defense Technology, No. 109 Deya Road, Kaifu District, 410086 Changsha, China; College of Computer Science and Electronic Engineering, Hunan University, 116 Lushan South Road, Yuelu District, 410086 Changsha, China

**Keywords:** molecular representation learning, foundation models, drug discovery, machine learning

## Abstract

Molecular representation learning (MRL) is afoundation in leveraging computational methods for drug discovery, enabling the transformation of molecular structure and properties into numerical vectors. These vectors serve as input for machine learning models and facilitate the prediction and analysis of molecular attributes, functions, and reactions. The advent of foundation models has introduced both new opportunities and challenges to MRL. These models have improved generalizability and migration in scarce data. Through pretraining and fine-tuning, foundation models can be adapted to various domains. Their robust encoding and generative abilities also allow the transformation of molecular data into more expressive forms. This paper provides a detailed review of current mainstream molecular descriptors and datasets, focusing primarily on the representation of small molecules while excluding larger molecules such as proteins and peptides. It classifies foundation models into two primary categories based on the form of input: unimodal-based and multimodal-based models. For each category, representative models are identified and their advantages and disadvantages evaluated. Moreover, we systematically summarize four core pretraining strategies for MRL foundation models, analyzing their task designs, applicable scenarios, and impacts on downstream performance. In addition, the application of molecular representation foundation models in drug discovery and development is discussed, together with the current status of model interpretability. The paper concludes with insights into the future directions of MRL foundation models.

## Introduction

With the rapid advancement of artificial intelligence, deep learning applications in real-world scenarios have attracted growing attention across diverse scientific domains. In particular, in chemistry and medicine, these applications encompass molecular property prediction, drug–drug interaction (DDI) analysis, and molecular generation (MG) [1–4]. Fundamental to these advances are effective molecular representation learning (MRL) methods, which enable the capture of molecular characteristics at different levels [5]. MRL leverages deep learning models to transform molecular structures and properties into numerical vectors, primarily through sequence-based and graph-based representation frameworks.

Historically, molecular fingerprints were initially used to encode molecules as binary vectors for the input of models [6], but public databases exhibited limited characteristics suitable for this representation. The Simplified Molecular Input Line Entry System (SMILES) addressed some of these limitations, facilitating the use of sequence-based neural architectures (e.g. Transformers and Recurrent Neural Networks (RNNs)) for the prediction of molecular tasks [7, 8]. Subsequently, researchers began to try to represent molecules as topological graphs, with atoms as nodes and bonds as edges [9, 10]. More recently, 3D molecular geometry has gained traction in molecular representation, as it better captures spatial structure–function relationships and reveals unique energy states [11, 12].

**Figure 1:**
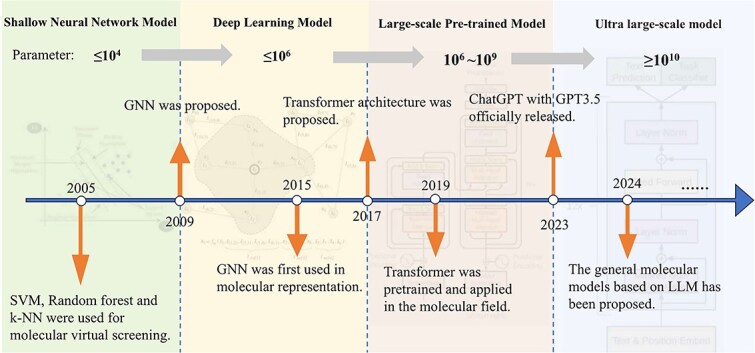
The development process of MRL foundation model.

Foundation models, defined as machine learning architectures characterized by massive parameter scales, enable adaptation to complex tasks [13–15]. Their development has progressed through four distinct stages: shallow neural network model, deep learning model, large-scale pretrained model, and ultra large-scale model with annual model size increasing 10-fold (from millions to billions of parameters) [16, 17], as illustrated in Fig. 1. Early computational resource constraints prioritized smaller architectures such as support vector machines (SVMs) [18], but advances in computational capacity and data scalability since 2009 have broadened neural network applications in molecular research [19, 20]. Since then, deep learning models have become ubiquitous in molecular representation tasks.

With increasing data volumes and computing resources, the scale of pretrained models has continued to grow, demonstrating robust generalization, and accuracy [21–24]. The advent of large language models (LLMs) has prompted increasing numbers of scholars to explore their applicability in molecular science. Combined with the availability of billion-level datasets in MRL [25], they are gaining traction in molecular modeling.

However, a systematic review of this field remains notably lacking. In this article, we investigate recent foundation models of MRL. Since foundation models in the molecular field are still in their early stages, most have significantly fewer parameters compared with LLMs. Thus, in the molecular field, we define models with millions of parameters as foundation models or large-scale models and summarize them to facilitate quick understanding for researchers new to the field and to aid in practical applications. Our contributions can be summarized in the following aspects:

(1) Categorization by inputs: We classify molecular representations on the basis of data-driven types. Using these categories, we outline learning strategies, application tasks, and representative foundation models associated with specific domain knowledge.
(2) Abundant additional resources: We have compiled a comprehensive collection of resources, including links to code repositories and benchmark datasets, to support further research and application.
(3) Guidance for choosing MRL foundation models: We present a comprehensive overview of MRL foundation models employed in each prevalent molecular task and systematically formulate a structured set of guidelines to assist in the judicious selection of appropriate models.
(4) Future outlook: We discuss the limitations of current models and highlight promising research directions that could lead to breakthroughs in the field.

## Molecular descriptors and datasets

**Figure 2:**
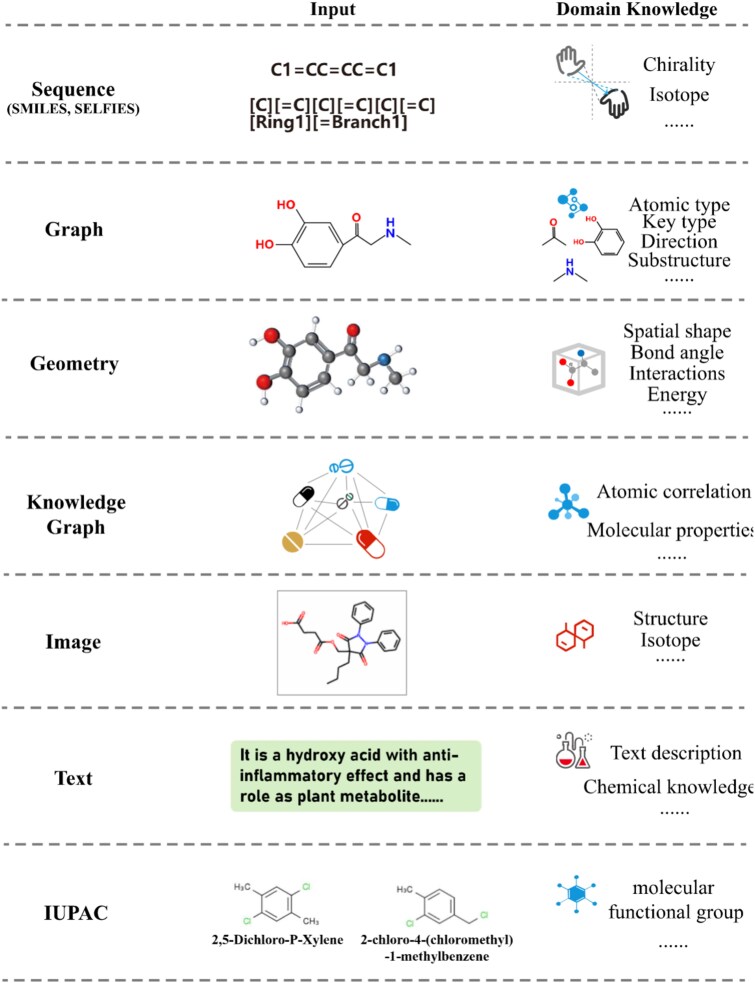
An overview of different inputs of MRL foundation models.

A typical foundation model framework involves pretraining the model using unlabeled large-scale datasets to learn generalizable representations, followed by fine-tuning on labeled downstream datasets [26]. Molecular descriptors, which vary in the type of information they provide, are crucial for this process. As depicted in Fig. 2, the utility of these descriptors depends on the specific downstream tasks. For example, 2D topological graphs generally reflect molecular size and degree of branching, which correlate with several drug properties, such as toxicity [27, 28]. In contrast, 3D geometric descriptors offer spatial information on atoms, conformational correlations, and surface properties, which are essential to determine quantum mechanical properties [29].

The success of pretraining foundation models is highly dependent on the underlying datasets, which are intimately linked to molecular representation techniques [30]. Numerous data are available, usually containing data on the biochemical properties and biological activities of molecules, such as those found in PubChem, ZINC, and GEOM [31–33]. We have detailed these data in Table 1, outlining their application tasks, data sizes, and other relevant information.

**Table 1:** Representative molecular datasets of foundation models

Dataset	Application tasks	Molecular descriptor	Data size	Link
PubChem	–	SMILES, 3D coordinates, Image, IUPAC names	$\sim $ 118 M	https://pubchem.ncbi.nlm.nih.gov
ZINC15	–	SMILES	$\sim $ 980 M	https://zinc15.docking.org
ChEMBL	–	SMILES	$\sim $ 2.4 M	https://www.ebi.ac.uk/chembl/
Chemical element KG	–	Knowledge graph	1643 Triples	https://github.com/ZJU-Fangyin/KCL/tree/main
PDBbind	PP, MG	SMILES	11 906	http://pdbbind.org.cn
BACE	PP, MG	SMILES	1512	https://deepchemdata.s3-us-west-1.amazonaws.com/datasets/bace.csv
BBBP	PP, MG	SMILES	2037	https://deepchemdata.s3-us-west-1.amazonaws.com/datasets/BBBP.csv
ClinTox	PP, MG	SMILES	1476	https://deepchemdata.s3-us-west-1.amazonaws.com/datasets/clintox.csv.gz
SIDER	PP, MG	SMILES	1425	https://deepchemdata.s3-us-west-1.amazonaws.com/datasets/sider.csv.gz
Tox21	PP, MG	SMILES	7830	https://deepchemdata.s3-us-west-1.amazonaws.com/datasets/tox21.csv.gz
ToxCast	PP, MG	SMILES	8574	https://deepchemdata.s3-us-west-1.amazonaws.com/datasets/toxcast_data.csv.gz
HIV	PP, MG	SMILES	41 125	https://deepchemdata.s3-us-west-1.amazonaws.com/datasets/HIV.csv
MUV	PP, MG	SMILES	93 086	https://deepchemdata.s3-us-west-1.amazonaws.com/datasets/muv.csv.gz
FreeSolv	PP, MG	SMILES	641	https://deepchemdata.s3-us-west-1.amazonaws.com/datasets/SAMPL.csv
ESOL	PP, MG	SMILES	1126	https://deepchemdata.s3-us-west-1.amazonaws.com/datasets/delaney-processed.csv
Lipo	PP, MG	SMILES	4200	https://deepchemdata.s3-us-west-1.amazonaws.com/datasets/Lipophilicity.csv
CEP	PP, MG	SMILES	29 978	http://www.cepii.fr/cepii/en/bdd_modele/bdd_modele.asp
QM7	PP, MG	SMILES	6830	https://deepchemdata.s3-us-west-1.amazonaws.com/datasets/qm7.mat
QM8	PP, MG	SMILES	21 787	https://deepchemdata.s3-us-west-1.amazonaws.com/datasets/qm8.csv
QM9	PP, MG	SMILES	133 884	https://deepchemdata.s3-us-west-1.amazonaws.com/datasets/qm9.csv
DrugBank	DDI	SMILES	612 386	https://github.com/xzenglab/KGNN
ZINC-250k	MG	SMILES	250 000	https://github.com/mkusner/grammarVAE
GEOM	PP, MG	3D coordinates	450 000	https://github.com/learningmatter-mit/geom
USPTO_MIT	RP	SMILES	950 000	https://github.com/wengong-jin/nips17-rexgen
USPTO-15K	RP	SMILES	15 000	https://github.com/connorcoley/ochem-predict_nn
USPTO-full	RP	SMILES	950 000	https://github.com/dan2097/patent-reaction-extraction
Molecule3D	PP, MG	3D coordinates	4 000 000	https://paperswithcode.com/dataset/molecule3d
PCQM4M-LSC	PP, MG	3D coordinates	3 378 606	https://ogb.stanford.edu/docs/lsc/pcqm4mv2/
ChemicalX DrugCombDB	DSP	SMILES	191 391	http://drugcombdb.denglab.org/download
DrugComb Portal	DSP	SMILES	718 002	https://drugcomb.org/download/

PP, property prediction; MG, molecular generation; RP, reaction prediction; DDI, drug–drug interaction; DSP, drug synergy prediction. “–” indicates not applicable. “$\sim $” means “about”. The first four datasets are pretraining datasets, so the application tasks are null.

We now briefly review the commonly used molecular descriptors and associated datasets.

Molecular fingerprint: This method converts molecular structures or properties into numerical or vector representations, widely used in computational chemistry and drug design for tasks such as virtual screening, similarity searches, and structure–activity relationship analysis. Morgan fingerprints, e.g. identify the presence or the absence of specific structures within molecules, generating binary bit-vector representations. This is done by considering the local environment of each atom, representing it based on a set of atomic invariants, and iteratively updating these features between adjacent atoms using a hash function. Such fingerprints are valued for their flexibility, compactness, and strong descriptive power [34, 35]. Various other types of molecular fingerprints include dictionary-based, circular, topological, pharmacophore, and protein–ligand interaction fingerprints [36].

1D sequences: It defines the string representation of molecules by performing a depth-first preorder spanning tree traversal of the molecular graph. The resulting string corresponds to the flattened spanning tree of the molecular graph. SMILES learning, noted for its compactness, has been extensively applied in molecular property prediction [37, 38]. SMILES strings explicitly represent meaningful substructures such as branching, cyclic structures, and chiral information. Due to its compatibility with computer programming, many datasets now utilize SMILES as molecular descriptors, including well-known repositories such as PubChem, ZINC, and others. However, the SMILES syntax is complex and highly restrictive, with most sequences not corresponding to well-defined molecules. Consequently, a new representation known as the SELFIES method was introduced by Krenn *et al.* [39] in 2020. This method aims to rectify prevalent grammar errors and violations of chemical principles in SMILES representations. SELFIES effectively solves these issues by associating each symbol with a specific structural or referential element, addressing common problems like imbalanced parentheses or ring identifiers. It can be converted from SMILES using various tools, enriching the available datasets.

2D Topology Graph: This method models atoms and bonds as nodes and edges, respectively, with each node and edge carrying feature vectors representing atomic type, chirality, bond type, and direction. Given that 2D molecular graph data can be derived from SMILES using the RDKit tool, possessing a SMILES dataset is effectively the same as having a 2D graph dataset [40]. Consequently, common SMILES datasets, such as those found in PubChem, ZINC, and others, can also serve as 2D graph molecular datasets. However, the inherent variability in topological structures often makes graph data more complex than image and text data, challenging the direct training of foundation models with molecular graphs.

3D Geometry: 3D geometry can display the arrangement of molecules in space, as well as the relative positions and directions between atoms, reflecting the spatial shape and stereochemistry of molecules [41]. Moreover, the energy state of molecules can be evaluated by calculating their potential energy, describing their interactions by calculating their molecular orbitals, and analyzing their dynamics by simulating their motion [42]. Structurally, stable molecules can be represented as a series of 3D coordinates [43]. However, 3D geometric data obtained through experimental measurements is typically costly, resulting in its scarcity in downstream tasks. To address this challenging issue, since September 2021, larger 3D datasets such as Molecule3D have been introduced. Models like GeoSSL are being pretrained on these datasets [44, 45].

Beyond the traditional molecular descriptors mentioned above, mathematically abstract molecular representations are also valuable for uncovering structural patterns and the chemical nature of molecules. These approaches quantify molecular features using tools from topology, graph theory, and differential geometry.

At the topological level, molecular structures can be modeled as topological spaces defined by atomic connectivity. Topological descriptors (e.g. Wiener index $W$) quantify structural complexity and predict stability and reactive site distribution [46]. It can be formulated as 


(1) \begin{align*}&W = \frac{1}{2} \sum_{i<j} d_{ij}\end{align*}

where $d_{ij}$ denotes the topological distance between atoms $i$ and $j$, defined as the number of edges in the shortest bonding path between them.

Graph theory provides computable models of molecular topology. Node centrality metrics (e.g. degree, betweenness, and closeness) quantify atomic importance, where high-degree atoms often indicate reactive centers. Degree centrality $C_{D}(v_{i})$ reflects local connectivity and is defined as: 


(2) \begin{align*}&C_{D}(v_{i}) = \sum_{j=1}^{n} A_{ij}\end{align*}

where $A \in \mathbb{R}^{n \times n}$ is the adjacency matrix of the molecular graph. $A_{ij} = 1$ indicates that atoms $v_{i}$ and $v_{j}$ are directly bonded; otherwise, $A_{ij} = 0$.

Betweenness centrality $C_{B}(v_{i})$ measures an atom’s mediation in molecular information transfer, indicating its influence on long-range interactions. Atoms with high betweenness centrality often serve as key intermediates. It is defined as: 


(3) \begin{align*}&C_{B}(v_{i}) = \sum_{s \neq i \neq t} \frac{\sigma_{st}(v_{i})}{\sigma_{st}}\end{align*}

where $\sigma _{st}$ denotes the total number of shortest paths between nodes $s$ and $t$, and $\sigma _{st}(v_{i})$ represents the number of those shortest paths that pass through node $v_{i}$.

Differential geometry methods capture molecular surface shape through curvature analysis, enabling characterization of geometric compatibility in drug–target binding [47]. Gaussian curvature $K$ and mean curvature $H$ describe local surface features (e.g. protrusions and cavities) relevant to binding complementarity, defined as: 


(4) \begin{align*} & K = \kappa_{1} \cdot \kappa_{2} \end{align*}

(5) \begin{align*} & H = \frac{\kappa_{1} + \kappa_{2}}{2} \end{align*}

where $\kappa _{1}$ and $\kappa _{2}$ are the two principal curvatures at any point on the molecular surface (with $K>0$ in convex regions, $K<0$ in concave regions, and $K=0$ on flat regions).

Such mathematically abstract representations help reveal the intrinsic relationships between molecular structure and properties, and provide more generalizable feature inputs for MRL models.

In addition to traditional molecular descriptors, recent research has explored the integration of data from various modalities with molecular representations, including IUPAC names, knowledge graphs (KGs), images, and biochemical texts. The IUPAC name, governed by rules from the International Union of Pure and Applied Chemistry, ensures that each chemical substance is uniquely named to accurately reflect its structure and composition, such as the position and type of functional groups. Molecular KGs amalgamate chemical knowledge with graph-structured data, linking chemical elements, molecular structures, and chemical reactions into a comprehensive network [48]. Furthermore, molecular images serve as another form of molecular descriptor, focusing on visual representation [49].

In addition, there is growing interest in the textual aspects of molecular data. Biomedical texts offer rich, flexible external information about molecular entities derived from wet lab experiments [50].

## Pretraining strategies

Pretraining strategies are core determinants of the performance of MRL foundation models. By designing rational pretraining tasks, these strategies enable models to learn generalizable molecular features from large-scale unlabeled molecular data, laying a solid foundation for downstream task fine-tuning. Currently, mainstream pretraining strategies for MRL foundation models can be categorized into four paradigms, with distinct task designs, applicable scenarios, and impacts on downstream performance, as elaborated below.

### Masked Language Modeling

Masked language modeling (MLM) serves as the cornerstone pretraining task for sequence-based MRL foundation models [51, 52]. The core logic of MLM involves randomly masking a subset of tokens in molecular sequences, followed by training the model to predict the masked tokens. This task forces the model to learn local dependency relationships and global sequence patterns between tokens, which is well suited to capturing the syntactic characteristics of molecular sequences.

For instance, ChemBERTa conducts MLM pretraining on 77 million SMILES sequences from PubChem [51]. By learning the correlations between atomic tokens and functional group tokens during pretraining, the model can accurately encode molecular structural features. Compared with traditional task-specific models, ChemBERTa achieves a 5%–10% improvement in AUC-ROC on molecular property prediction tasks. However, MLM has inherent limitations: it over-reliance on the syntactic correctness of sequences and fails to effectively capture spatial and topological features of molecules. Thus, it is more suitable for unimodal models with sequence inputs.

### Contrastive learning

Contrastive learning (CL) has emerged as a dominant pretraining strategy for multimodal MRL models by constructing positive–negative sample pairs to align features across different modalities or different views of the same modality. In unimodal scenarios, CL generates negative samples by perturbing molecular graphs and performs CL between original graphs and perturbed graphs, enhancing the robustness to molecular topological feature variations. In multimodal scenarios, CL aligns features from different modalities, enabling cross-modal information fusion.

GraphMVP exemplifies the effectiveness of CL in multimodal pretraining. By contrasting topological features of 2D molecular graphs with spatial features of 3D geometry, the model simultaneously captures molecular connectivity and spatiality. In the energy prediction task on the QM9 dataset, GraphMVP reduces the RMSE by 15% compared with unimodal models [53]. The key advantage of CL lies in its ability to learn inter-modal correlations without labeled data. However, its performance highly depends on the quality of positive sample construction, which remains a critical challenge in practical applications.

### Reconstruction-based pretraining

Reconstruction-based pretraining (RBP) enables models to learn global molecular structural features by reconstructing original molecular data from corrupted inputs. For graph-based models, reconstruction tasks include “node feature reconstruction” and “graph structure reconstruction” [54]. For 3D geometry-based models, reconstruction tasks involve “coordinate reconstruction” and “energy reconstruction” [55].

Molecular Graph Masked Autoencoder (MGMAE) demonstrates the superiority of RBP in graph models. By masking >50% of nodes and edges in molecular graphs and training the model to reconstruct complete molecular graphs, MGMAE forces the model to learn global topological patterns of molecules. On the BBBP dataset for molecular property prediction, MGMAE achieves an AUC-ROC of 94.2%, outperforming peer models [56]. The primary advantage of RBP is its ability to capture global molecular features, but it requires high model complexity and incurs relatively high training costs.

### Multimodal alignment pretraining

Multimodal alignment pretraining (MAP) is specifically designed for multimodal input models, aiming to align and fuse features from different modalities through cross-modal tasks [50, 57]. For example, KV-PLM adopts a “SMILES to text” matching task to align molecular structure and functional information [50].

The key advantage of MAP is its ability to fuse structural information (SMILES, graphs) and semantic information (text), providing more comprehensive features for downstream tasks. However, this strategy requires large-scale cross-modal labeled data, which poses significant challenges in data acquisition and annotation.

## Computing models

This section reviews prominent foundation models utilizing diverse molecular representation techniques (Table 2), where the “Molecular Descriptor” denotes pretraining inputs. Models are classified into Unimodal-based and Multimodal-based categories. Molecular fingerprints are rarely used due to information loss, dimensional complexity, and rule dependency [89, 90]. Instead, structure-based representation learning methods are preferred for preserving molecular information and enhancing model performance.

**Table 2:** Summary of representative molecular representation foundation models (MRFMs) from recent years

Model	Year	Molecular descriptor	Backbone architecture	Pretraining dataset	Parameters	Downstream Tasks	Link
ChemBERTa-2 [51]	2022	Sequence (SMILES)	Transformer	PubChem (77 M)	5 M–46 M	PP	–
MOLFORMER [52]	2022	Sequence (SMILES)	Bert	PubChem + ZINC15 ($\sim $1111 M)	$\sim $ 21 M	PP	https://github.com/IBM/molformer
MOLGEN [58]	2024	Sequence (SELFIES)	Bart [59]	ZINC15 (100 M)	8B	MG	https://github.com/zjunlp/MolGen
LlaSMol [60]	2024	Sequence (SMILES/SELFIES)	Mistral [61]	SMolInstruct (3 M)	7B	PP, MG, RP	https://github.com/osu-nlp-group/llm4chem
SynerGPT [62]	2024	Sequence (SMILES)	Transformer	ChemicalX DrugCombDB (0.6 M)	18 M/22.8 M	DSP	https://github.com/KyleBenzle/SynerGPT
CancerGPT [63]	2024	Sequence (SMILES)	GPT	DrugComb Portal (0.7 M)	124 M	DSP	–
GROVER [5]	2020	Graph	GNN + Transformer	ZINC15+Chembl (10 M)	107.7 M	PP	https://github.com/tencent-ailab/grover
GraphCL [64]	2020	Graph	5-layer GIN	ZINC15 (2 M)	$\sim $ 2 M	PP	https://github.com/Shen-Lab/GraphCL
Hu *et al.* [65]	2020	Graph	5-layer GIN	ChEMBL(456k)+ZINC(2 M)	$\sim $ 2 M	PP	http://snap.stanford.edu/gnn-pretrain
JOAO [66]	2021	Graph	5-layer GIN	ZINC15 (2 M)	$\sim $ 2 M	PP	https://github.com/Shen-Lab/GraphCL_Automated
AD-GCL [67]	2021	Graph	5-layer GIN	ZINC15 (2 M)	$\sim $ 2 M	PP	https://github.com/susheels/adgcl
GraphLoG [68]	2021	Graph	5-layer GIN	ZINC15 (2 M)	$\sim $ 2 M	PP	https://github.com/DeepGraphLearning/GraphLoG
MPG [69]	2021	Graph	MolGNet [70]	ZINC + ChEMBL (11 M)	53 M	PP, DDI	https://github.com/pyli0628/MPG
MGSSL [71]	2021	Graph	5-layer GIN	ZINC15 (250 K)	$\sim $ 2 M	PP	https://github.com/zaixizhang/MGSSL
Graphomer [72]	2021	Graph	Transformer	PCQM4M-LSC (3.8 M)	47.1 M	PP	https://github.com/microsoft/Graphormer
LP-Info [73]	2022	Graph	5-layer GIN	ZINC15 (2 M)	$\sim $ 2 M	PP	https://github.com/Shen-Lab/GraphCL_Automated
SimGRACE [74]	2022	Graph	5-layer GIN	ZINC15 (2 M)	$\sim $ 2 M	PP	https://github.com/junxia97/SimGRACE
GraphMAE [54]	2022	Graph	5-layer GIN	ZINC15 (2 M)	$\sim $ 2 M	PP	https://github.com/THUDM/GraphMAE
MGMAE [56]	2022	Graph	5-layer GIN	ZINC15 (2 M) + ChEMBL (456 K)	$\sim $ 2 M	PP	–
KPGT [75]	2022	Graph	Transformer	ChEMBL (2 M)	100 M	PP	https://github.com/lihan97/KPGT
MOLE-BERT [76]	2023	Graph	5-layer GIN	ZINC15 (20 M)	$\sim $ 2 M	PP	https://github.com/junxia97/Mole-BERT
3D PGT [77]	2023	3D Geometry	GPS [78]	PubChemQC (3.74 M)	42.6 M	PP	https://github.com/LARS-research/3D-PGT
Uni-Mol [55]	2023	3D Geometry	Transformer	ZINC/ChemBL + PDB (209 M)	$\sim $ 47.61 M	PP, MG	https://github.com/dptech-corp/Uni-Mol
ImageMol [49]	2022	Images	ResNet18 [79]	PubChem (10 M)	$\sim $ 11 M	PP	https://github.com/ChengF-Lab/ImageMol
MM-Deacon [80]	2021	Sequence + IUPAC	Transformer	PubChem	10 M	PP, DDI	–
PanGu Drug Model [81]	2022	Sequence + Graph	Transformer	ZINC20 + DrugSpaceX + UniChem ($\sim $1.7B)	104 M	-	http://pangu-drug.com/
DVMP [82]	2023	Sequence + Graph	GNN + Transformer	PubChem (10 M)	104.1 M	PP, RP	https://github.com/microsoft/DVMP
Transformer-M [83]	2023	Graph + 3D Geometry	Transformer	PCQM4Mv2 (3.4 M)	47.1 M	PP	https://github.com/lsj2408/Transformer-M
GraphMVP [53]	2022	Graph + 3D Geometry	5-layer GIN + SchNet	GEOM (50 K)	$\sim $ 2 M	PP	https://github.com/chao1224/GraphMVP
KV-PLM [50]	2022	Sequence + Text	Transformer	PubChem (150 M)	$\sim $ 110 M	PP	https://github.com/thunlp/KV-PLM
MolT5 [57]	2022	Sequence + Text	Transformer	ZINC-15 (100 M)	60 M / 770 M	MG	https://github.com/blender-nlp/MolT5
MoleculeSTM [84]	2023	Sequence + Text	GNN + Transformer + Bert	PubChemSTM (281K)	$\sim $ 400 M	PP, MG	https://github.com/chao1224/MoleculeSTM/tree/main
MolReGPT [85]	2024	Sequence + Text	GPT	ChEBI-20 (33 K)	$\sim $ 1T	MG	https://github.com/phenixace/MolReGPT
TxT-LLM [86]	2024	Sequence + Text	PaLM-2 [87]	TDC ($\sim $726 K)	$\sim $ 400 M	PP, RP	–
Y-mol [88]	2024	Sequence + Text + KG	Llama2	PubMed ($\sim $33 M)	7B	PP, DDI, MG	https://anonymous.4open.science/r/Y-Mol

PP, property prediction; MG, molecular generation; RP, retrosynthesis prediction; RC, retrosynthesis condition prediction; DDI, drug–drug interaction, DSP, drug synergy prediction. The symbol “–” indicates that a particular application is not applicable, and “$\sim $” denotes “approximately”.

### Unimodal-based model

#### Sequence-based model

SMILES-based models encode chirality via tokenization and pretraining design. Chiral centers marked by “@” and “@@” are treated as distinct tokens, allowing the Transformer’s self-attention to learn their contextual associations. For example, the “[C@@H]” token forms strong attention weights with nearby C, N, and O atoms, capturing the local chiral environment.

In addition, SELFIES-based representations explicitly encode chirality using semantically clear tokens such as “[C@]” and “[C@@]”, reducing syntactic complexity [91]. Their fragment-level tokenization strengthens the linkage between chiral information and the molecular backbone, enabling foundation models to learn chirality–property relationships more efficiently via positional encoding and dependency modeling.

Transformer models surpass early RNN-based approaches due to their superior ability to capture chemical structure information. RNNs suffer from gradient vanishing, limiting their capacity to model long-range dependencies, and nonlocal interactions critical for molecular properties. In contrast, Transformers employ self-attention to encode global token relationships, enabling effective representation of functional groups and their spatial correlations. This capability supports more accurate learning of structure–activity relationships.

Foundation models such as GPT, BERT, and T5, which are derived from Transformer architectures, have reshaped the trajectory of MRL. In chemical informatics, researchers have leveraged their capabilities, exemplified by ChemBERTa, which integrates chemical sequence representation [92]. These models have excelled in tasks like retrosynthesis and molecular property prediction, leading White to proclaim that “the future of chemistry is language” [93].

A significant challenge with SMILES-based models is the generation of invalid molecular strings. To mitigate this, models must incorporate additional rules, such as SMILES syntax and atomic ordering, which complicate training [94]. Alternatively, Selfies sequences, as adopted by MolGEN, offer a solution to this problem [58].

#### Topological graph-based model

Molecular graph representation is increasingly recognized for its ability to better capture the structural and functional characteristics of molecules compared with sequence inputs [95]. Topology-based graph models leverage message passing and graph structural encoding to exploit molecular topological features. The common method involves using graph neural networks (GNNs) [96], graph convolutional networks (GCNs) [97], graph attention networks (GATs) [98], and graph isomorphism networks (GINs) [99] for molecular representation. They stack multiple graph convolution layers and integrates atomic features (node features) with bond features (edge features). Through “aggregation and update” operations, it learns the local topological environment of each node. For example, when updating node representations, GIN aggregates the features of all neighboring nodes and uses learnable parameters to adjust their contribution weights, enabling the model to capture branching complexity, ring structures, and other topological characteristics.

However, traditional GNNs are limited by message passing that only covers immediate neighboring nodes, making it difficult to capture nonlocal chemical interactions. In contrast, Transformer-based graph models overcome this limitation through global self-attention, enabling them to encode potential relationships between any pair of atoms.

Similar to sequence models, Transformer-based graph models (e.g. Graphormer) overcome traditional GNN’s nonlocal interaction limitation to capture topological features [72]. Through this architectural design, Graphormer efficiently leverages molecular topological features and achieves superior performance in molecular property prediction tasks compared with conventional GNNs.

#### 3D geometry-based model

Most MRL methods represent molecules as sequential tokens or topology graphs, limiting their ability to leverage 3D geometry essential for 3D-related tasks. Suboptimal 3D structure parameters can degrade model performance compared to sequential/topological methods, and may also cause robustness and prediction issues. Overcoming this in foundation models remains a major challenge [100].

3D GNN-based models extract local spatial features of atoms through convolutional operations. However, their fixed receptive fields limit the ability to adapt to dynamic conformational changes in molecules. In contrast, Transformer-based 3D models employ distance-aware self-attention, which dynamically adjusts attention weights between atoms to capture key spatial interactions in real time as conformations evolve.

Taking Uni-Mol as an example, the model first converts the 3D coordinates of atoms into a relative Euclidean distance matrix. Through the self-attention modules of a prelayerNorm Transformer, distance information is incorporated into attention weight computation—atoms that are closer in space receive higher attention weights [101], while long-range interactions are effectively captured via distance thresholding and attention weight adjustment [55, 102, 103].

In addition, Uni-Mol introduces an SE(3)-equivariant coordinate head, which preserves invariance to spatial rotations and translations during training, ensuring robust generalization of spatial shape representations [77].

3D PGT employs a graph processing system (GPS) [104] architecture that integrates 3D coordinates with molecular graph topology. By using spatial convolution layers, it extracts local atomic spatial distribution features and fuses them with graph attention mechanisms, enabling joint encoding of molecular spatial geometry, and chemical environment.

#### Image-based model

Advances in computer vision have led to growing interest in image-based MRL. Although both molecular images and topological graphs serve as 2D representations, they differ in processing paradigms: GNNs model graph connectivity and chemical topology, whereas CNNs extract local visual patterns from images. Recent progress in unsupervised visual representation learning [105, 106] suggests strong potential for image-based pretraining in drug discovery. For example, ImageMol [49] introduces a chemistry-aware unsupervised framework that achieves high accuracy across multiple drug discovery tasks, demonstrating the utility of molecular images as an effective representation modality.

Traditional unimodal molecular descriptors, such as fingerprints, SMILES strings, and 2D topological graphs, show strengths in specific tasks but inherently suffer from limited information capacity. As MRL applications expand toward more complex scenarios, information from a single modality is no longer sufficient to support a comprehensive understanding of molecular characteristics. Consequently, multimodal molecular descriptors have emerged. The core advantage of multimodal inputs lies in their ability to integrate complementary information from different modalities, thereby enabling more holistic and informative molecular representations.

### Multimodal-based model

In recent years, the advancement of foundation models has notably improved the understanding and generation capabilities of multimodal data. These models use extensive multimodal datasets to learn associations between different modalities, thereby enhancing their ability to interpret and generate such data. When trained on diverse datasets, the representations developed by foundation models are highly transferable, offering broad applicability across numerous downstream tasks. This universality has substantial research value, especially in the field of drug discovery, where it may potentially lead to significant breakthroughs. In the following, we will introduce four distinct types of multimodal integration.

#### Sequence+Graph integration

While SMILES sequences alone may not adequately capture the topological structure of molecules, relying solely on molecular graphs can lead to issues such as overly smooth graph models. Employing both SMILES and molecular diagrams simultaneously allows for the leveraging of each method’s strengths, achieving a more comprehensive representation of molecular structures. However, current approaches, such as the DVMP, whose dual-tower architecture lacks fine-grained interaction between SMILES and graph data, which is addressed by MolCLR’s CL [82, 107].

#### Graph+3D geometry integration

While graphs primarily emphasize topological information, 3D geometry focuses on energy-related features. Molecular graphs effectively capture the composition and chemical structure of molecules, where atoms and bonds are represented as nodes and edges [108]. In contrast, 3D geometry displays the spatial arrangement of molecules, showing relative positions and directions among atoms. It can reveal the molecular spatial shape and stereochemistry [41]. Together, they can facilitate a deeper analysis of reaction rates and pathways. GraphMVP exemplifies this approach by enriching 2D topological pretraining with 3D geometric information, which provides robust supplementary information on molecular energy and spatial structure data [53].

Due to the limitations inherent in 3D geometric data, it is often necessary to combine 3D information with 2D data for model input. A notable approach in this area is GeomGCL, which uses a geometric CL strategy on both 2D and 3D views [109]. Currently, it improves the MRL in 2D by incorporating additional 3D geometric information. This dual-view strategy not only solves the integration challenges, but also improves the overall predictive performance of the models.

Despite these advancements, the scarcity of comprehensive molecular datasets that include 2D and 3D information remains a significant challenge, limiting the development of foundation models in this field.

#### Text+Other representation integration

In recent years, a new trend in molecular representation has focused on jointly modeling molecular SMILES sequences with literature texts to obtain cross-modal representations. The combination of SMILES structural information and textual context provides stronger prior knowledge for tasks, such as molecular property prediction and domain knowledge extraction, especially useful for fine-tuning LLMs on chemical tasks.

KV-PLM initiated the integration of biochemical text with SMILES sequences, where the model first converts SMILES sequences into 2D molecular graphs via RDKit, then embeds the 2D molecular graph features (e.g. atomic type and bond type) into the text encoding process through a cross-modal attention module, thereby facilitating cross-modal learning between molecular structure and biochemical text [50]. MolT5 further developed this concept with a pretrained model handling large volumes of unlabeled text and molecular strings [57].

Subsequent models like MoleculeSTM use CL to align molecules and text for zero-shot retrieval and editing [84]; MolReGPT bridges molecular and natural languages via retrieval-augmented prompting [85].

Despite these advancements, this approach inherits limitations of SMILES and NLP. Textual data often focus on either internal molecular structures or external biomedical contexts, restricting machine reading versatility and impacting knowledge acquisition and pretrained model performance.

#### Other multimodal methods

Beyond conventional descriptors, molecular information can also be represented through images, KGs, and other modalities [48–50]. These developments have motivated multimodal representation learning, which integrates heterogeneous data to provide a more comprehensive molecular understanding. For example, KCL unifies structural features with knowledge associations to uncover latent inter-element relationships [48]. Many recent frameworks adopt separate encoding branches for different data types, such as DVMP for structural strings and topological graphs, MM-Deacon for chemical naming semantics, and CLOOME for integrating molecular and cellular imaging signals [80, 82, 110]. The resulting embeddings are jointly optimized to establish cross-modal correspondence.

However, these models require large amounts of single-modal data and complex cross-modal alignment, increasing pretraining cost, and limiting scalability.

## Applications

**Figure 3:**
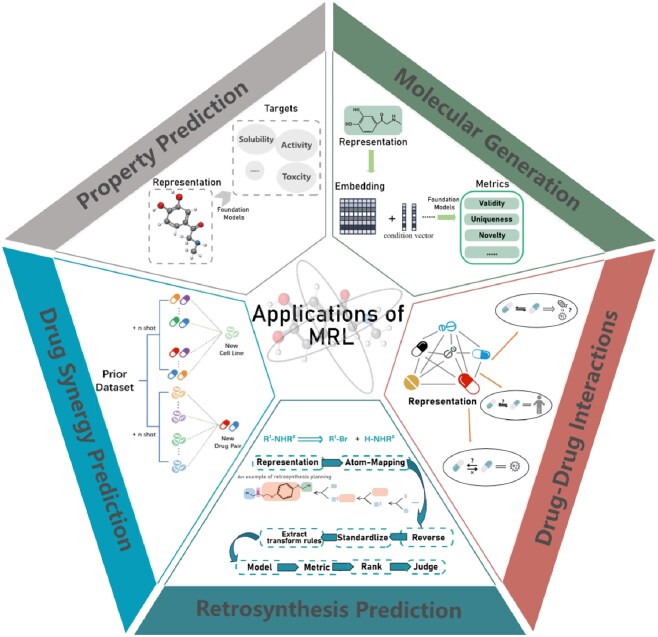
Applications of MRL foundation model.

This section explores four prevalent application tasks of MRFMs. Each task is pivotal in leveraging the potential of these models within various domains as depicted in Fig. 3.

We detail representative works for each application in Table 2, providing a comprehensive overview of the methods and their outcomes. In Table 2, “Downstream tasks” include those specified in the original studies; however, the models may also be applicable to other tasks not listed.

### Application 1: molecular property prediction

Molecular property prediction is vital in fields like drug development [111, 112], involving the prediction of molecule physical and chemical properties from structural data. The accuracy of these predictions hinges on learning effective molecular representations, making property prediction a common performance benchmark for MRL foundation models.

AUC-ROC and RMSE/MAE are the two main evaluation metrics used for classification and regression tasks, respectively. As shown in Table 3, foundation models integrating Transformer- or GIN-based architectures excel in molecular property prediction. Notably, Chemprop, a pivotal model based on Directional Message Passing Neural Networks (D-MPNNs), has significantly advanced molecular property prediction by introducing directional message passing mechanisms [113]. This innovation addresses the limitation of traditional MPNNs in ignoring bond directionality, enabling more accurate capture of asymmetric electronic effects and steric hindrance. Chemprop achieved state-of-the-art performance, and served as a key inspiration for subsequent advanced models like CD-MVGNN [114] and GSL-MPP [115]. With the emergence of Transformer architectures, their combination with GNNs has been applied to molecular property prediction tasks and has shown remarkable performance. DVMP, with its dual-branch Transformer and GNN architecture, shows strong performance in molecular property prediction, especially on the HIV and SIDER datasets [82].

**Table 3:** Comparison of performance (ROC-AUC %) on molecular property classification tasks

	BBBP	BACE	ClinTox	Tox21	ToxCast	SIDER	HIV	MUV
ChemBERTa-2	64.3	–	73.3	72.8	–	–	62.2	–
MOLFORMER	93.7	88.21	94.8	84.7	–	69	**82.2**	–
GROVER	94	89.4	94.4	83.1	73.7	65.8	–	–
GraphCL	69.68	75.38	75.99	73.87	62.4	60.53	78.47	69.8
Hu *et al.*	68.7	84.5	72.6	78.1	65.7	62.7	79.9	81.3
JOAO	71.39	75.49	80.97	74.27	63.16	60.49	77.51	73.67
AD-GCL	69.54	77.27	80.77	72.92	–	63.19	–	–
GraphLoG	72.5	83.5	76.7	75.7	63.5	61.2	77.8	76
MPG	92.2	92	96.3	83.7	74.8	66.1	–	–
MGSSL	69.7	79.1	80.7	76.5	64.1	61.8	78.8	78.7
Graphomer	–	–	–	–	–	–	80.51	–
LP-Info	71.68	81.15	76.73	74.45	62.39	60.8	77.03	72.03
SimGRACE	71.3	75	75.6	75.6	63.4	60.6	75.2	76.9
GraphMAE	72	83.1	82.3	75.5	64.1	60.3	77.2	76.3
MGMAE	**94.2**	**92.7**	96.7	86	75.3	66.4	–	–
KPGT	90.8	85.5	94.6	84.8	74.6	64.9	–	–
MOLE-BERT	71.9	80.8	78.9	76.8	64.3	62.8	78.2	78.6
3D PGT	72.1	80.9	79.4	73.8	69.2	60.6	78.1	69.4
Uni-Mol	72.9	85.7	91.9	79.6	69.6	65.9	80.8	**82.1**
MM-Deacon	78.5	–	**99.5**	–	–	69.3	80.1	–
DVMP	77.8	89.4	95.6	79.1	–	**69.8**	81.4	–
GraphMVP	72.4	81.2	77.5	74.4	63.1	63.9	77	75
KV-PLM	74.61	–	–	72.71	–	61.51	74	–
MoleculeSTM	69.98	80.77	92.53	76.91	65.05	60.96	76.93	73.4
TxT-LLM	–	–	86.3	**88.2**	**79.2**	–	73.2	–

The best performing values are highlighted in bold, and the second best ones are marked with an underline; “–” indicates no data available. All performance metrics in this table are directly cited from the original studies of the corresponding models, and the evaluation protocols are consistent with the original studies to ensure the reliability of performance comparison.

However, model effectiveness depends heavily on the dataset. Mole-BERT encounters negative transfer due to a small and unbalanced atomic vocabulary [76]. While techniques like VQ-VAE can alleviate this, challenges may resurface in tasks such as protein prediction, indicating the need for alternative strategies.

### Application 2: molecular generation

In drug discovery, identifying target molecules with specific properties, historically relying on domain expertise, poses a significant challenge. MG automates this process via four steps: (i) Data conversion: molecule structures are transformed into computational formats (e.g. SMILES, graphs, andvectors). (ii) MG: deep learning-based generative models sample or create novel molecules from the molecular space. (iii) Molecular evaluation: molecules are screened according to predicted properties, including physical, chemical, biological, toxicity, and synthesizability aspects. (iv) Molecule optimization: generated molecules are refined, or model parameters adjusted, to improve metrics such as QED, SA, and pIC50 [116–118].

**Table 4:** Comparison of performance on molecule generation tasks

Model	Dataset	Validity$\uparrow $ (%)	Novelty$\uparrow $ (%)	COV$\uparrow $ (%)	MAT$\downarrow $ (Å)	FCD$\downarrow $
LlaSMol (using SELFIES)	SMolInstruct	99.9	–	–	–	–
MOLGEN	Synthetic molecules	100	100	–	–	0.15
	Natural product molecules	100	99.87	–	–	65.19
Uni-Mol	QM9	–	–	97.95	0.1831	–
	Drugs	–	–	91.91	0.7863	–
MolT5	CheBI-20	90.5	–	–	–	1.2
MolReGPT (10-shot)	ChEBI-20	89.9	–	–	–	0.41
Y-mol	Random samples (200 000)	100	68	–	–	–

Validity: chemical validity; Novelty: The percentage of valid molecules generated that do not appear in the training dataset; COV, coverage; MAT, average atomic distance; FCD, Frechet ChemNet Distance. “$\uparrow $”: The larger the numerical value, the better the performance; “$\downarrow $”: The smaller the numerical value, the better the performance; “–” indicates no data available. All performance metrics in this table are directly cited from the original studies of the corresponding models, and the evaluation protocols are consistent with the original studies to ensure the reliability of performance comparison.

For evaluating MG, two primary criteria are often used: (i) validity: the proportion of chemically viable molecules out of all generated molecules [119]. (ii) Novelty: the percentage of valid molecules generated that do not appear in the training dataset.

Additional metrics include: (i) reconstruction accuracy: measures how frequently the model can reconstruct a specific molecule from its potential embeddings [120]. (2) FCD: assesses the similarity between sampled and training molecules [121]. (3) For tasks with attribute constraints, the proportion of generated molecules that match the target attribute is also evaluated.

In tasks like molecular conformation generation, coverage score (COV) and matching score (MAT) are commonly used metrics to assess performance [122].

As shown in Table 4, foundation models like MolT5 and Uni-Mol have proven effective in MG. MolT5, pretrained on natural language and SMILES, excels at generating molecules from text descriptions, outperforming baselines in validity. Uni-Mol generates 3D molecular conformations, achieving superior COV and MAT scores compared to other models [55, 57].

### Application 3: drug–drug interactions

DDI tasks play a pivotal role in the drug development process, aiding drug developers in screening for safer and more effective drugs. They also assist clinicians in making informed decisions and arranging appropriate treatment plans, thereby enhancing drug safety, reducing healthcare costs, and minimizing medical disputes [123].

In the realm of DDI prediction, several metrics are commonly employed to assess the performance of models: (i) accuracy (ACC): measures the proportion of correct predictions made by the model. (ii) ROC–AUC (AUC): evaluates the model’s ability to discriminate between interacting and noninteracting drug pairs. (iii) PR-AUC (Area Under Precision-Recall Curve): focuses on the precision and recall performance of the model, particularly useful in datasets with class imbalances. (iv) F1 Score: Balances precision and recall, providing a measure of the model’s accuracy in identifying true interactions.

As shown in Table 5, MPG, a specialized foundation model for DDI prediction [69], conducts unsupervised pretraining on large molecular datasets to obtain general molecular representations. Then, it refines these representations via supervised fine-tuning on a smaller labeled dataset to learn specific molecular pair interactions. Finally, MPG uses a multitask learning strategy to predict interaction types and degrees simultaneously, enhancing DDI prediction comprehensiveness and accuracy.

**Table 5:** Comparison of performance of AUC-ROC(%), PR-AUC(%), and F1(%) on DDI tasks

Model	Dataset	AUC-ROC (%)	PR-AUC (%)	F1 (%)
MPG	BIOSNAP	96.6	96	90.5
MM-Deacon	Zhang’s dataset [124]	95	91.8	82.14
Y-mol	Ryu’s dataset [125]	65.23	–	–
	Deng’s dataset [126]	62.19	–	–

The higher the value of AUC-ROC, PR-AUC, F1, the better the performance is; “–” indicates no data available. All performance metrics in this table are directly cited from the original studies of the corresponding models, and the evaluation protocols are consistent with the original studies to ensure the reliability of performance comparison.

### Application 4: retrosynthesis prediction

Molecular representation plays a crucial role in retrosynthesis, which involves devising viable synthetic routes for target molecules [127]. This process benefits significantly from various molecular representation techniques that help chemists identify key substructures, select appropriate bond-breaking points, and predict potential synthons and precursors. Additionally, these techniques assist in validating reaction outcomes and products.

Each molecular representation method provides unique insights that are critical for retrosynthesis: (i) Molecular mass and elemental composition: Helps in determining the basic framework of the target molecule. (ii) Functional groups and stereochemistry: Crucial for understanding reactivity and orientation in molecular interactions. (iii) Crystal structure and intermolecular interactions: Aid in predicting how molecules will interact under different conditions.

By integrating this information, chemists can design more rational retrosynthetic pathways, optimize reaction conditions, and enhance both the efficiency and selectivity of the synthesis process. As shown in Table 6, DVMP is a foundational model that has demonstrated robust performance in retrosynthesis tasks. After undergoing pretraining with a vast dataset, it effectively supports the complex decision-making required in planning and executing synthetic routes [82].

**Table 6:** Comparison of performance (top-k Accuracy %) tested on USPTO-50K of the retrosynthesis task

Model	Top-k accuracy(%)					
	1	3	5	10	20	50
DVMP (Reaction types unknown)	54.2	70.5	77.2	84.9	90	92.7
DVMP (Reaction types give as prior)	**66.5**	**81.2**	**86.6**	**90.5**	**92.8**	**93.5**
TxT-LLM	23.9	–	–	–	–	–

The best performing values are highlighted in bold, and the second best ones are marked with an underline; “–” indicates no data available. All performance metrics in this table are directly cited from the original studies of the corresponding models, and the evaluation protocols are consistent with the original studies to ensure the reliability of performance comparison.

The most prevalent metric in retrosynthesis analysis is the accuracy of top k (Top-k ACC), which measures the proportion of correctly predicted retrosynthesis routes among the first k predictions.

### Application 5: drug synergy prediction

Drug synergy prediction assesses whether combined drug effects are synergistic or antagonistic, crucial for cancer, antimicrobial therapy, and complex disease management. MRL transforms drug structures into vectors and constructs joint embeddings for prediction.

Foundation models have been applied to this task, mainly through transfer learning with pretrained models or fine-tuning LLMs. These approaches overcome data scarcity, performing well even with limited datasets.

Common evaluation metrics include ROC-AUC, PR-AUC, accuracy (ACC), and the F1 score. ROC-AUC evaluates model discrimination, PR-AUC is useful for imbalanced datasets, ACC measures prediction correctness, and the F1 score balances precision and recall.

As shown in Table 7, CancerGPT uses LLMs to predict synergy in rare tissues, outperforming others in PR-AUC and ROC-AUC in multiple datasets [63] in particular. SynerGPT, a GPT-based model, applies contextual learning to personalized prediction, achieving high ROC-AUC scores (74.0 in zero-shot and 77.7 in few-shot) in novel drug combinations, surpassing baselines [62].

**Table 7:** Comparison of performance of AUC-ROC (%) and PR-AUC (%) on drug synergy prediction tasks

Model	Dataset	AUC-ROC (%)	PR-AUC (%)
CancerGPT	DrugComb portal	–	–
SynerGPT (zero-shot)	DrugCombDB-unknown drug	74	57.3
	DrugCombDB-unknown cell line	83.5	72.1
SynerGPT (few-shot)	DrugCombDB-unknown drug	**77.7**	**61.5**
	DrugCombDB-unknown cell line	**83.8**	**72.8**

The best performing values are highlighted in bold; “–” indicates no data available. CancerGPT has not released specific AUC-ROC and PR-AUC data publicly. All performance metrics in this table are directly cited from the original studies of the corresponding models, and the evaluation protocols are consistent with the original studies to ensure the reliability of performance comparison.

### How to choose the appropriate foundation model

When applying molecular foundation models to downstream tasks, researchers should consider the following strategic selection guidelines.

First, effective model selection in MRL requires systematic consideration of task objectives, data characteristics, and architectural constraints. Molecular property prediction prioritizes accurate estimation of physicochemical or biological properties, whereas MG emphasizes creating novel yet chemically valid structures. Retrosynthesis focuses on decomposing targets into feasible precursors, while DDI and synergy prediction require modeling combinatorial pharmacological effects.

Second, data properties further guide model choices. SMILES- or graph-based representations are well suited for MG, where Transformer architectures or graph variational autoencoders (GVAE) [128] are commonly adopted. Retrosynthetic prediction requires precise mapping between reactants and products, making multimodal architectures advantageous. Interaction and synergy prediction benefit from capturing latent relational knowledge, where KG-enhanced models are preferred. For data-limited scenarios, transfer learning and fine-tuning of pretrained models (e.g. ChemBERTa-2 [51]) can improve performance.

Third, model selection should align with established paradigms. Predictive tasks—including property prediction and interaction modeling—can be handled by conventional GNNs, but large pretrained frameworks, such as ChemBERTa-2 [51] or Uni-Mol [55] offer greater accuracy and efficiency. Generative tasks typically rely on GPT-style autoregressive models [129], exemplified by MOLGEN [58]. Retrosynthesis lies at the interface of prediction and generation, requiring task-specific architectural trade-offs.

Finally, when interpretability is required, Transformer-based architectures should be prioritized due to their explainability through attention mechanisms. Attention matrix analysis can be incorporated to facilitate interpretability experiments when needed. For applications with training time constraints, simple architectures are recommended, because hybrid architectures typically require more training time, as shown in Table 8.

**Table 8:** Comparison of time complexity of MRFMs

Architecture	Model	Time complexity
GIN-based	GraphCL	$O(|V| + |E|)$
	Hu *et al.*	
	JOAO	
	AD-GCL	
	GraphLoG	
	MGSSL	
	LP-Info	
	SimGRACE	
	GraphMAE	
	MGMAE	
	MOLE-BERT	
Transformer-based	ChemBERTa-2	$O(n^{2} \cdot d)$
	SynerGPT	
	KPGT	
	Uni-Mol	
	MM-Deacon	
	PanGu drug model	
	Transformer-M	
	KV-PLM	
	Graphomer	
	MolT5	
GPT-based	CancerGPT	$O(n^{2} \cdot d)$
	MolReGPT	
Llama-based	Y-mol	$O(L \cdot (n^{2} \cdot d + n \cdot d^{2}))$
Ber-based	MOLFORMER	$O(n^{2} \cdot d)$
Bart-based	MOLGEN	$O(n^{2} \cdot d)$
Mistral-based	LlaSMol	$O\left (L \cdot \left (n \cdot w \cdot d + n \cdot d^{2}\right )\right )$
GPS-based	3D PGT	$O(n^{2} \cdot d + |E|)$
ResNet18-based	ImageMol	$O(k^{2} \cdot c)$
PaLM-2-based	TxT-LLM	$\mathcal{O}\left (L \cdot \left (n^{2} \cdot d + n \cdot d^{2}\right )\right ) $
MolGNet-based	MPG	$O(|V| + |E|)$
GNN + Transformer-based	GROVER	$O(|V| + |E| + n^{2} \cdot d)$
	DVMP	
GIN + SchNet-based	GraphMVP	$O(|V| + |E| + k^{3})$
GNN + Transformer + Bert-based	MoleculeSTM	$O(|V| + |E| + n^{2} \cdot d)$

The time complexity of GNN/GIN-type models is $\mathcal{O}(|V| + |E|)$, where $|V|$ represents the number of nodes in the graph structure and $|E|$ denotes the number of edges. The computational complexity of Transformer-based models is $\mathcal{O}(n^{2} \cdot d)$, where $n$ denotes the sequence length, and $d$ represents the embedding dimension. $L$ denotes the number of layers. In the time complexity of LlaSMol, $w$ represents the sliding window size. In the complexity formulas of ImageMol and GraphMVP, $k$ indicates the number of atoms.

## Interpretability

Interpretability is essential for identifying and mitigating model biases, ensuring fairness, and enhancing user trust in model predictions [130]. However, the complexity of foundation models—characterized by massive parameter scales and opaque internal mechanisms—limits transparency and controllability [131], underscoring the need for effective interpretability techniques [132].

Existing interpretability methods can be broadly categorized into three categories:

(1) Feature attribution methods quantify contributions of input features to model outputs, including gradient-based, surrogate-based, and perturbation-based approaches. Yet, the high dimensionality of modern molecular representations substantially increases computational cost and complexity [133].
(2) Instance-based methods explain predictions by analyzing specific samples through anchors [134], counterfactuals [135], or contrastive reasoning [136]. Their adoption remains limited due to the intensive computation required for generating valid counterfactuals or contrasts [133].
(3) Graph-convolution-based methods leverage message passing and attention mechanisms to assign importance weights to molecular graph components. Attention maps have become the most widely used interpretability strategy in foundation model research, with approaches, such as GNNExplainer [137] and DVMP [82]. While these methods capture complex relational structures, they incur substantial computation and memory overhead on large or deep networks, and may suffer from feature over-smoothing in deeper layers.

## Conclusion and future outlooks

MRL has greatly enhanced the efficiency and quality of molecular design, discovery, and optimization [138]. In this work, we first reviewed commonly used molecular descriptors and datasets, and categorized foundation models based on their input representations. For each category, representative MRL models were analyzed with respect to their architectural characteristics and performance trade-offs.

We further synthesized four mainstream pretraining paradigms for MRL foundation models, highlighting their modality-specific strengths and applicability to diverse downstream tasks. Additionally, we examined major applications of MRL models in drug discovery and assessed the progress and remaining issues in interpretability.

Despite the considerable advances achieved by MRL models, several challenges and limitations persist that warrant further investigation.

### Integrating multimodal data

The choice of molecular representation fundamentally shapes model input structure and determines the type of chemical information encoded, with each paradigm exhibiting distinct strengths and limitations. Sequence-based 1D representations are easy to implement but neglect spatial configuration [139]. Graph-based 2D methods capture atomic connectivity yet omit conformational details [140]. While 3D geometric models provide full spatial information, they must handle issues, such as conformational variability and rotational invariance [81]. Additionally, emerging modalities, such as molecular videos [141] and audio [142], suggest opportunities for richer multimodal characterization.

If these multimodal data are fused, it may be possible to represent molecules in a more comprehensive way for downstream tasks. To fully unlock the potential of multimodal fusion, future research should advance along three practical directions.

First, incorporating molecular dynamics (MD) trajectories as a novel dynamic modality can significantly enhance representational capacity. Unlike static 3D structures, MD trajectories capture continuous conformational transitions (e.g. ligand binding and unbinding events). Leveraging spatiotemporal attention mechanisms—even hybrid architectures combining 3D CNNs with Transformers—enables extraction of time-resolved features such as bond angle fluctuations and conformational transition kinetics. This helps address a key limitation of static structural models, which often overlook dynamic binding processes, thereby improving predictions of kinetic parameters such as k_on and k_off.

Second, cross-modal data augmentation provides a scalable solution for the scarcity of experimentally derived 3D structures. Abundant 2D graphs or SMILES representations can be used to pretrain generative models that propose physically plausible 3D conformations. These conformations can then be refined and filtered using chemical priors—such as bond length and valence constraints encoded in KGs—before being utilized to augment training datasets for 3D MRL models. Recent advances in MRL demonstrate that CL is effective for cross-modal representation alignment. For example, the GraphMVP framework integrates contrastive objectives with reconstruction tasks to jointly pretrain 2D and 3D molecular encoders [53].

Third, KG-guided multimodal alignment introduces chemically meaningful constraints during pretraining. By embedding chemistry-aware relational rules as alignment guidance, fused multimodal representations can remain consistent with fundamental chemical principles. This strategy is expected to reduce chemically invalid outputs and improve the reliability of generative modeling. These methodologies typically involve three fundamental components: (i) extracting entities and relationships from heterogeneous sources, (ii) performing cross-modal alignment and feature fusion, and (iii) constructing KGs and embedding them into a vector space for downstream tasks. Demonstrated systems such as AliMe MKG [143] validate the effectiveness of this framework, achieving performance gains in recommendation by integrating textual, visual, and user-generated content.

### Technologies for addressing data scarcity

The advancement of foundation models critically depends on access to extensive pretraining datasets. The limited availability of molecular data constitutes a major obstacle in developing robust MRFMs. To overcome these challenges, several technological approaches have shown effectiveness [144–146].

Semi-supervised learning offers a viable solution for 3D MRL in scenarios with scarce labeled data. A dual-task training paradigm can be adopted: large amounts of unlabeled 3D conformations (e.g. derived from MD simulations) are used for self-supervised pretraining—such as conformation denoising by perturbing atomic coordinates and reconstructing the original structure—while a smaller subset of experimentally validated 3D structures with annotated properties is used for supervised fine-tuning. Notably, this hybrid learning strategy has achieved >90% of the performance of fully supervised models using only 10% labeled data in 3D property prediction tasks on QM9 [147].

Cross-modal data augmentation can leverage the abundance of 2D molecular representations to alleviate 3D data sparsity. Methods such as 3D InfoMax enable the extraction of latent 3D structural information from 2D molecular graphs and the generation of multiple plausible 3D conformers for each input molecule. These generated conformations can then be screened and refined using chemistry-aware constraints, ensuring structural validity while substantially expanding the scale of 3D training datasets without requiring additional experiments.

Beyond these methodologies, analogous strategies have been developed, including meta-learning and knowledge distillation techniques. Meta-learning enables models to rapidly adapt to new tasks leveraging prior experience [148]. Knowledge distillation facilitates the transfer of knowledge from larger models to compact architectures without significant performance degradation [149].

### Interpretability

Despite substantial performance improvements in molecular foundation models, interpretability remains a key challenge [150]. While prior work has emphasized predictive accuracy, the growing complexity of MRL models now necessitates stronger interpretability to ensure reliable decision-making.

Current interpretability research in MRL primarily relies on attention-based visualization of key graph nodes, which is insufficient for fully explaining multimodal learning behavior. Future directions include assessing decision consistency across modalities to identify potential biases—e.g. discrepancies in feature focus between SMILES-based and 3D models. Existing tools, such as DODRIO and Align-Anything, support attention visualization within Transformers and across modalities, enabling more comprehensive interpretability analyses [151, 152].

Incorporating chemical KGs provides another promising avenue. By integrating structured domain knowledge into model architectures and subsequent interpretability evaluation, knowledge-guided analysis can better elucidate chemically meaningful reasoning processes.

### Efficiency of training

Foundation models demonstrate high accuracy and generalization in MRL and downstream tasks. However, their large parameter count demands processing vast data during training and inference, which consumes significant storage. Thus, efficient data management and transmission, such as distributed parallel training techniques, are essential [153, 154].

Data parallelism, a common approach, evenly distributes data across GPUs or nodes, differentiates gradients per device, aggregates them on one GPU, and broadcasts results [155, 156]. Future parallel computing advancements will likely boost foundation model training efficiency, streamlining processes for faster iterations, and complex model development, reducing current high-cost barriers.

### Robustness and generalization

Robustness and generalization are essential for reliable deployment of MRL models. Robustness ensures stable performance under perturbations or distribution shifts, while generalization enables effective prediction on unseen molecular domains.

Enhancement strategies operate at both the data and model levels. Data augmentation improves robustness by expanding distributional diversity, such as using multiple SMILES representations, generating 3D conformers via molecular simulations, or applying perturbations to atomic and bond features. Multimodal integration with contrastive alignment further strengthens cross-domain transferability [157].

At the algorithmic level, meta-learning approaches improve rapid adaptation to limited data [158]. Sparse attention mechanisms in Transformer architectures reduce sensitivity to irrelevant long-range interactions [159]. Additionally, probabilistic weighting techniques such as Monte Carlo Dropout [160] help mitigate noise and improve predictive reliability.

**Key Points**

- Provide the first systematic review of molecular representation learning (MRL) foundation models, with a focus on small-molecule representation techniques.
- Categorize MRL foundation models into unimodal and multimodal architectures and analyze representative models in each category.
- Summarize four mainstream pretraining strategies, highlighting their applicability, and influence on downstream molecular property prediction and generation tasks.
- Discuss challenges in model interpretability and provide practical guidelines for selecting appropriate MRL foundation models in real-world drug discovery scenarios.
- Propose future research directions, including multimodal fusion, data scarcity solutions, efficiency improvements, and robustness enhancement.

## Data Availability

All the code and data tables used for the figures in the manuscript are available on GitHub link: https://github.com/Z-dot-max/MRL_Foundation_Review/.
